# Endurance training slows breast tumor growth in mice by suppressing Treg cells recruitment to tumors

**DOI:** 10.1186/s12885-019-5745-7

**Published:** 2019-06-04

**Authors:** Amit Hagar, Zemin Wang, Sachiko Koyama, Josua Aponte Serrano, Luma Melo, Stephanie Vargas, Richard Carpenter, John Foley

**Affiliations:** 1 History & Philosophy of Science & Medicine Department, Indiana University, Morrison Hall 314, Bloomington, IN 47405 USA; 2 Intelligent Systems Engineering Department, Indiana University, Bloomington, IN USA; 3 Environmental Health Department, School of Public Health, Indiana University, Bloomington, IN USA; 4 Medical Sciences Program, Indiana University School of Medicine, Bloomington, USA; 5 Indiana University Cancer Center Indiana University School of Medicine, Indianapolis, USA; 6 Department of Dermatology, Indiana University School of Medicine, Indianapolis, USA

**Keywords:** Endurance exercise, Forced running wheels, Treg cells, CD8^+^/FoxP3^+^ ratio, Solid tumor progression, Murine mammary tumor, Hypoxia

## Abstract

**Background:**

Aerobic exercise has been shown to slow tumor progression in rodents and humans, but the mechanisms behind this effect are still unclear. Here we show that aerobic exercise in the form of chronic endurance training suppresses tumor recruitment of FoxP3^+^ Treg cells thus enhancing antitumor immune efficiency.

**Methods:**

Adult wild-type and athymic BALB/c female mice were endurance-trained for 8 weeks. Circulating leukocytes as well as muscle and liver mtDNA copy number were compared to aged-matched concurrent sedentary controls to establish systemic effects. 4 T1 murine mammary tumor cells were injected subcutaneously to the 4th mammary pad at the end of the training period. Tumor growth and survival rates were compared, together with antitumor immune response.

**Results:**

Exercised wild-type had 17% slower growth rate, 24% longer survival, and 2-fold tumor-CD^+^ 8/FoxP3^+^ ratio than sedentary controls. Exercised athymic BALB/c females showed no difference in tumor growth or survival rates when compared to sedentary controls.

**Conclusions:**

Cytotoxic T cells are a significant factor in endurance exercise-induced suppression of tumor growth. Endurance exercise enhances antitumor immune efficacy by increasing intratumoral CD8^+^/FoxP3^+^ ratio.

**Electronic supplementary material:**

The online version of this article (10.1186/s12885-019-5745-7) contains supplementary material, which is available to authorized users.

## Background

Aerobic exercise has been known to systemically alter many physiological features in humans [1] and is currently considered a part of “standard of care” approaches to the prevention of many common chronic conditions [2]. The mechanisms that underlie its effect on the progression of disease, however, are harder to assess given the obvious limitations on adherence of patients to structured physical exercise and the difficulty of obtaining dose-response data in the critically ill. To circumvent this limitation in human patients, rodent exercise models have been developed with the two most prevalent being the voluntary running wheel [3] and the electric-shock treadmill [4].

Both these models, however, are suboptimal for the purpose of mechanistic evaluation of dose-response effects in human disease models. While the voluntary running wheel does not force the exercise on the animals, unless proper monitoring technology is deployed it requires stress-inducing single-caging so that individual dose recordings can be made for each animal. The problem here is that since the running is voluntary, the dose is not controllable, and varies widely between animals. In addition, given mice behavioral patterns, the type of aerobic exercise this model simulates is closer to high intensity interval training (HIIT) rather than to chronic endurance exercise (CEE) [5]. Both HIIT and CEE have similar results on improving aerobic fitness [6], but untrained humans prefer to pursue the latter rather than the former, as it is easier to implement on a regular continuous basis [7]. In addition, mice perform voluntary running in dosage that no human, even elite athletes, can mimic with HIIT, which limits the human relevance of the voluntary wheel model. The other prevalent model, the electric-shock treadmill, can be used to induce CEE and allows group caging, but is based on forcing an unmotivated animal to continue exercising with an electric shock grid. Therefore, it may incur additional physical stress that may mask desired health effects. As a result, only a small fraction of exercise rodent models mimic controllable and quantifiable aerobic exercise in dosages that are relevant to human disease [8].

Based on epidemiologic evidence and randomized exercise intervention trials on the association between physical activity and breast cancer risk [9], the role of physical activity in breast cancer prevention is widely acknowledged today. Studies show average reduction in breast cancer risk in physically active women, with the strongest associations found for recreational and household activities and for activity that was of at least moderate intensity and sustained over a lifetime [10]. More recently, a pilot study in post-menopausal women detected a correlation between tumor progression rates of early stage invasive ductal carcinoma and the aerobic fitness levels of the hosts: the more aerobically fit is the patient, the longer is her estimated tumor doubling time [11]. Motivated by this phenomenon, the purpose of the pilot study presented here was to investigate potential mechanisms behind it in mice.

The well-known systemic effects of aerobic exercise on the immune system [12] form a natural basis for this investigation. The intricate relations between solid tumors and the immune system have been a subject of ongoing research [13], with a recent rise in interest due to the rediscovery of the immunotherapeutic paradigm [14]. Cytotoxic T cells are an effective tool in the host’s battle against solid tumors, but since the late 1970s it was suggested that T cells are also capable of suppressing the rejection of implanted tumors [15]. Two decades later it was demonstrated that CD4^+^FoxP3^+^ T-cells, termed “regulatory,” or Tregs, were responsible for the induction of dominant immune tolerance to tumors [16]. Their ability to inhibit antitumor response, quantified by the intratumoral ratio between CD8^+^ cytotoxic T cells and FoxP3^+^ Treg cells, has been shown to be a marker for antitumor immunity [17]. A relatively high number of FoxP3^+^ Treg cells, resulting in a decreased CD8^+^/FoxP3^+^ ratio, is also significantly associated with shorter overall survival in the majority of solid tumors investigated, including breast cancer [18–20].

In this pilot study we introduce a controllable and quantifiable rodent model for aerobic exercise as an alternative to the standard treadmill or the voluntary running wheel that avoids single caging or electrical shock. We also suggest a low-stress CEE training protocol with which we identify a minimal dosage of endurance training sufficient for inducing systemic changes in circulating leukocytes and markers for mitochondrial function. Applying this exercise model to a murine mammary tumor, we were able to detect a significant suppression of tumor growth in animals that underwent endurance training compared to sedentary mice. We further found that tumor growth suppression was lost in T cell-deficient mice suggesting the effects on immune function are a significant factor in exercise-induced suppression of tumors.

## Methods

### Running wheel apparatus

The model is based on two parts, a set of forced running wheels and a controllable moving belt on which they can be mounted (Additional file 1: Figure S1). This apparatus houses 4 mice, one per wheel, and in principle can be used to control and quantify endurance training for an individual mouse. For the pilot experiment detailed below, we used 3 apparatuses and calibrated their digital controllers so that training of 12 mice can be performed simultaneously at the same running speed.

### Animals

The training protocol conformed to the standards of humane animal care and was approved by the Indiana University Bloomington IACUC. Twenty-four 10-week-old BALB/c female mice and sixteen age-matched Foxn1−/− nude (athymic) BALB/c female mice (Charles River Laboratory) were housed four per cage in a 12 h/12 h light/dark cycle, with mean temperature of 23 ± 2 °C and relative humidity of 55 ±10 %. Mice were divided randomly to two groups, Exercised (Ex) and Sedentary (Sed). Mice were fed standard chow and water ad libitum, and their individual weight and mean daily food intake were monitored once a week by measuring cage food weight in two consecutive days, ignoring nibbling waste in the bedding. Before sample collection, mice were initially anesthetized by 2% isoflurane supplied by a vaporizer and exsanguinated with a cardiac left ventricle stick. Death was confirmed by cervical dislocation.

### Training protocol

In the training period mice exercised 5 days a week, for 8 consecutive weeks. Prior to this period, in the first 2 weeks, mice were acclimatized to the running apparatus, spending 5 min inside the wheels without running in week 1, and then 8 min with minimum velocity (2 m/min) in week 2. From the 1st week of the training period onwards they increasingly spent more time running, starting at minimum velocity and increasing it in the last 2 min of the run to the maximum velocity of that week. This maximum velocity increased throughout the training period. (Additional file 1: Figure S2).

To avoid stress, we trained the mice with no a-priori goal. Instead, we implemented the following rule: when a mouse would show first signs of exhaustion by freezing or clinging to the rungs, the velocity would be lowered until the mouse would begin running again. This rule ensured the mice kept running continuously for longer and longer periods with slowly increasing velocities, adjusting the intensity level to the ability of the lowest performing mouse. In the 8th and final week the mice ran for 26 min a day, spending 1 min at 6 m/min, 1 min at 8 m/min, 22 min at 10 m/min, and 2 min 12 m/min.

To eliminate other potential sources of difference between the groups and to isolate training effects as the sole factor for the systemic changes, the sedentary mice were put inside the wheels immediately after the running session has ended for an increasing equivalent time period without the wheel being activated. Feces were collected from cages of both groups before, during, and after the training period to control for cortisol levels.

After training period ended, mice remained in their cages for 72 h to avoid transient effects. At that point 6 mice were picked randomly, 3 from each group, and were sacrificed. The remaining mice were used for tumor inoculation. No statistical difference in mean weight was detected between each random sample and the respective original group (2-sided t-test p_Ex_ = 0.58 and p_Sed_ = 0.49, respectively).

### Lactate kinetics

To further validate the training protocol, we have repeated it in same-age 6 female Balb/c mice and performed a variation of a lactate threshold test on them immediately after training period has ended. In this experiment blood lactate concentration was measured from a tail-prick using a handheld blood lactate analyzer (Lactate+, Nova Biomedical). Measurements were performed before exercise (base level), immediately after a 5 min session has ended in the wheel with 8 m/min running speed, and then in two relaxation time points 5 min and 15 min after the exercise has ended. 6 same-age sedentary Balb/c females were used as control group.

### Tumor inoculation and size measurements

4 T1 murine mammary tumor cell line (ATCC) was used for inoculation. 2 × 10^4^ cells were injected subcutaneously into the 4th mammary pad. 2 out of 9 exercised wild-type BALB/c mice had to be discarded due to injection failure. Tumor volume was measured with a caliper by the same person who was blinded to the study groups. Once tumor volume exceeded 1000 mm^3^ mice were sacrificed and blood, tumor and tissue were harvested.

### qPCR and gene expression

Hind limb muscles (from the rear right leg) and livers were collected and snap frozen in liquid N_2_. Total RNA was collected from muscle using an RNA isolation system (Promega). Genomic DNA were collected from muscle and liver tissue using a DNA purification kit (Promega). Total RNA from muscle was subjected to reverse transcription (RT) followed by qPCR with the following primers: Tnni1 (Forward): 5′-CCACGAGGACTAAACTAGGCA-3′, Tnni1 (Reverse): 5′-CCTCTCAACTTCCGGCATGG-3′; Tnni2 (Forward): 5′-CCGCCGAGAATCTGAGAAGG-3′, Tnni2 (Reverse): 5′-TGCAGAGTTCCTGCACTTCA-3′; ActinB (Forward): 5′-CACTGTCGAGTCGCGTCC-3′, ActinB (Reverse): 5′-CGCAGCGATATCGTCATCCA-3′. Muscle and liver mtDNA was subjected to qPCR using the following primers: CoxII (Forward): 5′-GCCGACTAAATCAAGCAACA-3′, CoxII (Reverse): 5′-CAATGGGCATAAAGCTATGG-3′; D-loop (Forward): 5′-GGCCCATTAAACTTGGGGGT-3′, D-loop (Reverse): 5′-GGCTGATTAGACCCGATACCA-3′; BetaGlobin (Forward): 5′-GAAGCGATTCTAGGGAGCAG-3′, BetaGlobin (Reverse): 5′-GAGCAGCGATTCTGAGTAGAGA-3′.

### Fecal corticosterone measures

Thirty fecal pellets were randomly collected from cages of exercised and sedentary mice prior to, and during training at the 1st, 4th, 7th, and 10th week. Fecal pellets were initially frozen and one day prior to extraction were dried 16 h at 60 °C then homogenized extracted in ethyl acetate (0 .1 ml/.1 g) with vigorous shaking for 30 min. The suspension was spun at 2500 rpm for 30 min in a swinging bucket rotor in the Allegra 6R centrifuge (Beckman Coulter), the supernate removed, evaporated with a positive pressure manifold (Biotage) and stored at -20 °C. The reside was dissolved in 100ul absolute ethanol (Koptec), then two 5ul samples taken, diluted with 4 volumes of AB buffer from the kit (Corticosterone Elisa, Arbor Assays) and vortexed. Elisa was performed according to kit instructions.

### Cell blood counts (CBC)

Blood was collected into heparinized vials via intracardiac puncture. Whole blood samples were subjected to standard CBC via ANTECH Diagnostics (Indianapolis) that included white blood cell, neutrophil, lymphocyte, monocyte, and eosinophil counts.

### Immunohistochemistry

Formalin-fixed, paraffin-embedded tumor tissue from the mice tumors were subjected to IHC as previously described [21, 22]. The antibodies used included CD8a and FoxP3 (Cell Signaling Technology).

### Muscles dissection

A combination of Soleus and Gastrocnemius muscle fibers were dissected from the right hind limb of each animal.

### Statistical analysis

Data were expressed as mean ± SE and were compared with commercial SPSS software using two-sided student t-test between independent means and, given the small sample size in some of the tests, a non-parametric mean difference permutation test which has no assumptions of distribution. Survival curves and log rank estimates were obtained with commercial SPSS software. *p* < 0.05 was considered statically significant.

## Results

### An established low-stress CEE model

**Fig. 1:**
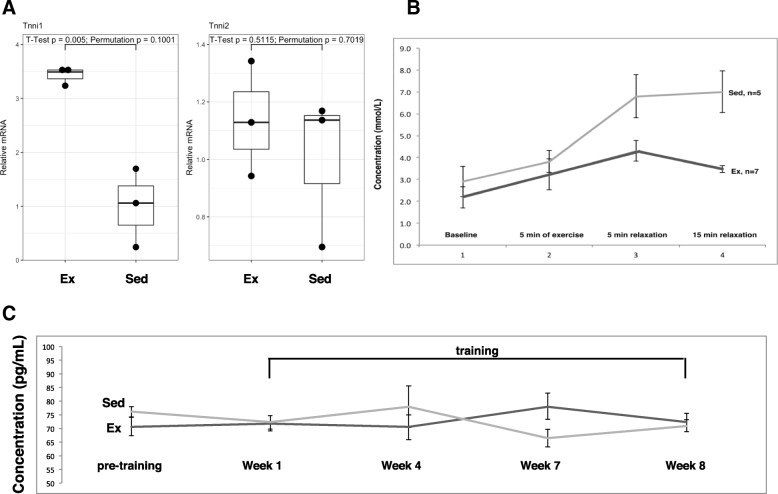
An established low-stress CEE model. **a** Effect of  8 weeks endurance training on slow twitch muscles composition. NS = statistically not significant. Data presented as mean ± SE with 95% confidence intervals. **b** Lactate kinetics after short exercise bout. Lactate levels measured in 0.7 *μL* blood drawn from tail with a handheld lactate analyzer (Lactate+ Nova-biomedical) showed faster clearance and lower concentration in endurance trained vs. sedentary adult Balb/c female mice (*N* = 6 in each group). **c** Mean cortisol levels (pg/mL) in feces collected from cages before, during and after training period. No significant difference was detected between the means (*n* = 16, *p* = 0.97), as well as between means at the different time points (*p* > 0.29 in all comparisons). Data presented as mean ± SE with 95% confidence intervals

8-weeks training on the model (following a 2-weeks orientation period) significantly increased slow twitch muscles expression, evidenced by increased *Tnni1* mRNA, but not fast twitch muscles, evidenced by no difference in *Tnni2* mRNA (Fig. 1a). This muscle type differential is a known marker for endurance fitness [23, 24]. We concentrated on a combination of soleus and gastrocnemius muscles as they are known to contain both slow and fast twitch fibers. In this pilot study we didn’t differentiate between them. Studies are underway to do so in a larger sample of mice. Additionally, we observed decreased mtDNA copy number in the liver (Additional file 1: Figure S3), a known marker for high-intensity endurance fitness level [25–27]. Finally, the lactate kinetics test showed that trained mice had lower levels of blood lactate after a short exercise period, and a quicker clearance rate than sedentary mice (Fig. 1b), a phenomenon that indicates greater aerobic fitness [28]. No significant differences between the exercise and the sedentary groups were detected with respect to food intake (Additional file 1: Figure S4). Lastly, cortisol levels measured from feces taken prior to, during, and at the end of the training period remained stable, and no difference was detected between the exercise and the sedentary groups (Fig. 1c), suggesting the exercise training program did not induce undue systemic stress on the animals. A systemic decreased level of circulating leukocytes was observed in the WBC differential analysis (Additional file 1: Figure S5). These effects were achieved with a maximal speed of 12 m/min at the 8th week, which is consistent with other reports on the maximal speed a mouse can continuously run without adverse stimuli [29]. The data indicate our novel murine exercise model and training protocol result in known physiological changes associated with vigorous endurance exercise training.

### Effect of training on tumor progression rate

**Fig. 2:**
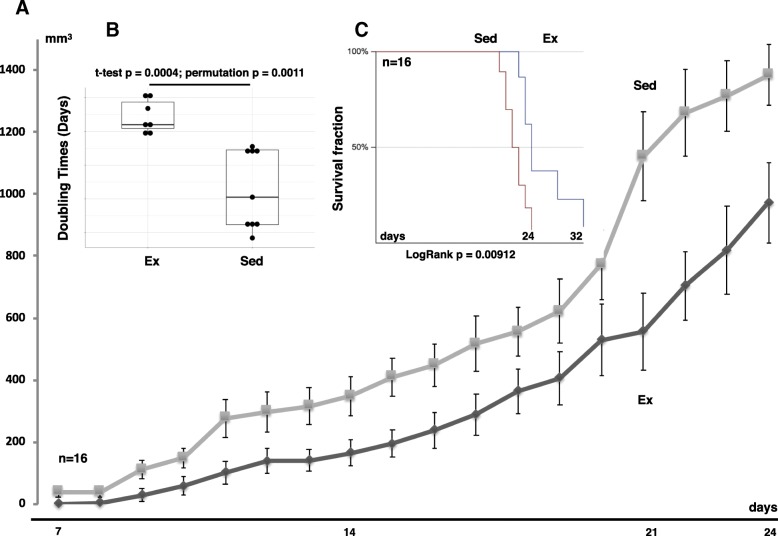
Effects of training on tumor growth and survival times in wild type mice. **a** Tumor growth curves for exercise trained (Ex, *n* = 7) and sedentary (Sed,  *n* = 9) groups. Data presented as mean ± SE with 95% confidence intervals. **b** Mean tumor growth rates (doubling times in days). Data presented as mean ± SE with 95% confidence intervals. **c** Kaplan-Meier curve showing significant difference in survival rates

To ascertain the effect of endurance training on early stage tumor growth, BALB/c mice were subjected to either the exercise program described above for 8 weeks or were kept sedentary as described in the Materials and Methods. 72 h after training had ended 4 T1 breast cancer cells were implanted in the mammary fat pad of exercised and sedentary mice. Mean tumor size in the exercised mice was significantly smaller than tumors in the sedentary animals throughout the time course of the study (Fig. 2a). The tumors in sedentary mice (*n* = 9) had a mean doubling time of 2.1 days ± 0.06 whereas the exercised mice (*n* = 7) had a mean doubling time of 2.46 days ± 0.11. A statistically significant difference in doubling time between the groups (Fig. 2b) was detected. A Kaplan Meier survival curve showed that exercised mice had a significantly longer survival compared to sedentary mice (Fig. 2c, Log rank *p* < 0.001). Furthermore, a significant difference was detected in mean survival days with the sedentary group at 21.3 days ± 0.5 and the exercised group at 25.1 days ± 1.4. These data strongly point to a slower tumor growth and an improved outcome for mice that were exercise-trained compared to sedentary mice.

### Endurance exercise results in a greater tumor immune response

**Fig. 3:**
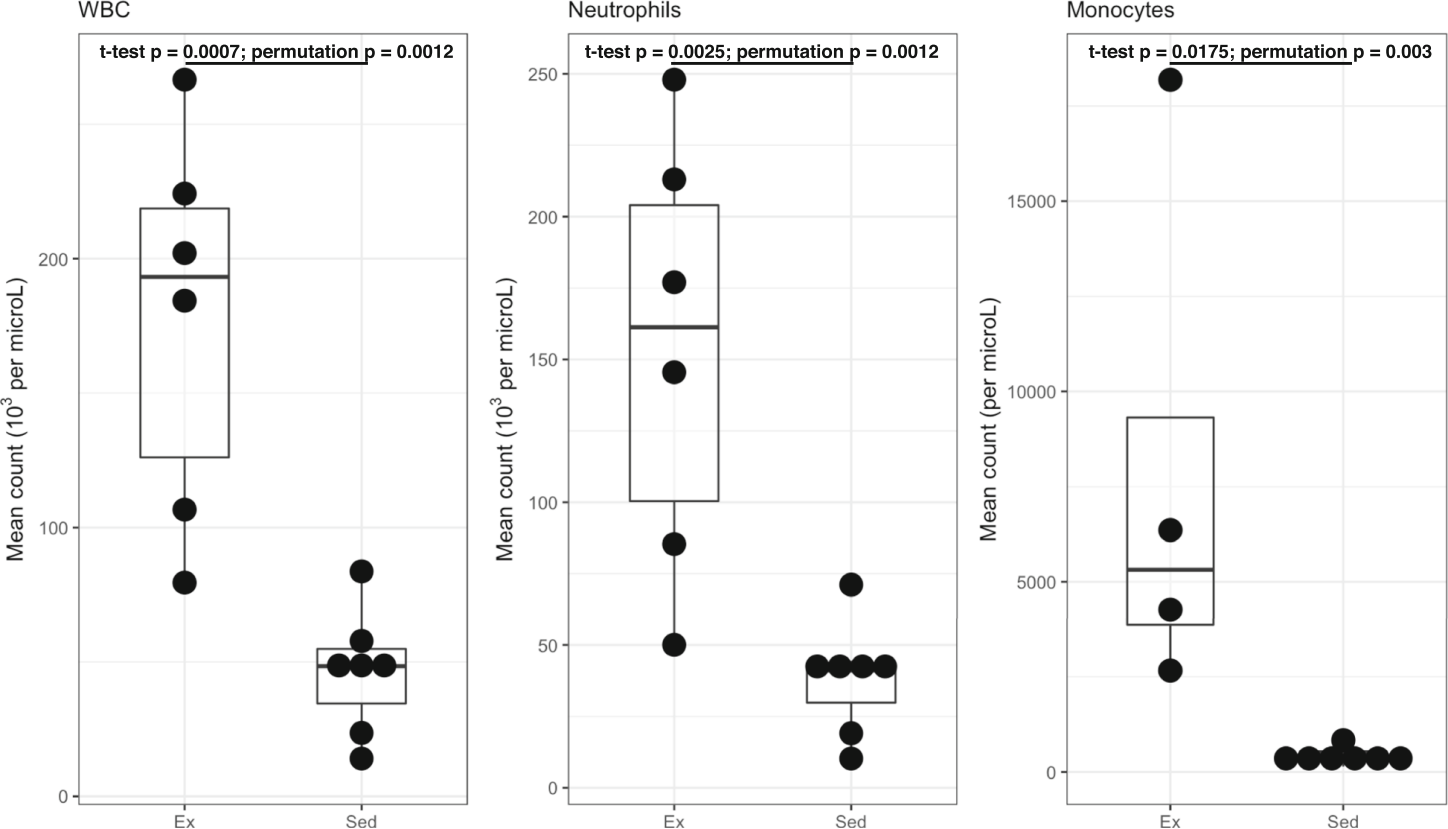
Effect of endurance training on adaptive immune response to 4 T1 tumor inoculation. Cells were injected subcutaneously to 4th mammary pad and grown until tumor size was>1000mm^3^. Exercise group (*n* = 7) had a significantly higher WBC (10^3^ / *μ L*), absolute neutrophil and monocytes counts (per *μL*) than the sedentary group (*n* = 9) with respective 2-sided t-test and mean difference permutation test *p* values as shown. Data presented as mean ± SE with 95% confidence intervals

In an attempt to understand a potential mechanism by which exercise training could have suppressed tumor growth and enhanced animal survival, the systemic immune response was assessed. Cell blood counts were performed on blood from the animals at the conclusion of the study. Absolute counts of circulating leukocytes were observed to be significantly lower in the exercise-trained group prior to tumor inoculation (Additional file 1: Figure S5), a phenomenon consistent with known systemic effects of CEE on the human immune system [30]. However, there was a significantly greater induction of an immune response in the exercised group in response to the presence of the tumor (Fig. 3 & Additional file 1: Figure S6), also consistent with prior evidence [31]. Total white blood cell count, neutrophils, and monocytes were significantly higher in systemic blood from the exercise-trained mice relative to the sedentary mice (Figs. 3a-c). These data would suggest a possible enhanced antitumor immune response in the exercise-trained mice.

### Endurance exercise enhanced antitumor immunity by increasing the intratumoral CD8^+^/FoxP3^+^ ratio

**Fig. 4:**
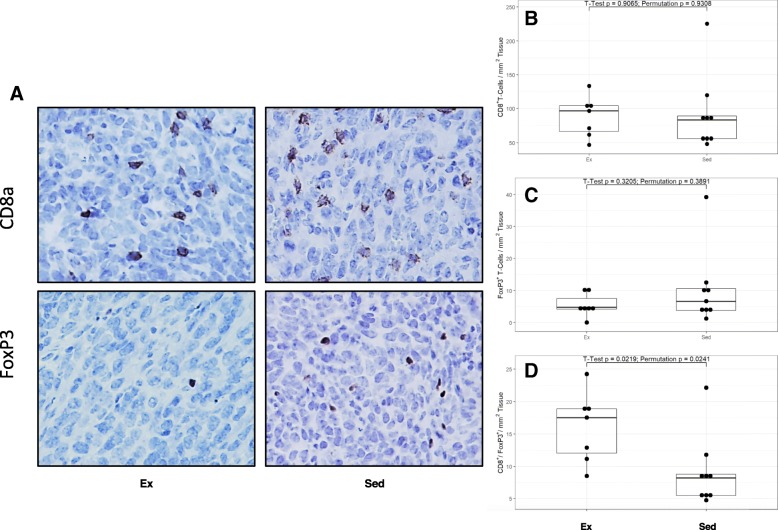
Intratumoral CD8^+^ and Treg cells. **a** IHC slides show difference in FoxP3^+^ densities between Sedentary (Sed) and Exercise (Ex). **b**-**d** Quantification of IHC results for CD8^+^ and Treg FoxP3^+^ inside tumor. Wild type groups (*n* = 16) showed no difference in density of positive cells (count per mm^2^) for both antibodies but showed a statistical difference in ratio between exercised (*n* = 7) and sedentary (*n* = 9) groups with p values as presented. NS = statistically not significant

To assess whether there was an increased antitumor immune response in the exercised mice, tumors were collected and subjected to IHC for the detection of T cells (Fig. 4a). The presence of T cells, specifically the intratumoral ratio between CD8^+^ cytotoxic T cells and FoxP3^+^ Treg cells, has been previously shown to be a marker for antitumor immunity [15]. Furthermore, a relatively high number of FoxP3^+^ Treg cells, resulting in a decreased CD^+^ 8/FoxP3^+^ ratio, is also strongly associated with poor prognosis in breast cancer patients [18–20]. Interestingly, significantly lower levels of FoxP3^+^ Tregs were observed in the tumors from exercised mice compared to sedentary tumors (Fig. 3b). No difference in CD8^+^ T cells were observed in the tumors from exercised and sedentary mice (Fig. 3c). However, the change in FoxP3^+^ cells resulted in a significantly higher CD8^+^/FoxP3^+^ ratio in exercise trained animals (Fig. 3d). The data suggest exercised animals have a greater antitumor immunity that could account for the observed suppression of tumor growth and enhanced survival.

### No effect on tumor growth in immune deficient athymic mice

**Fig. 5:**
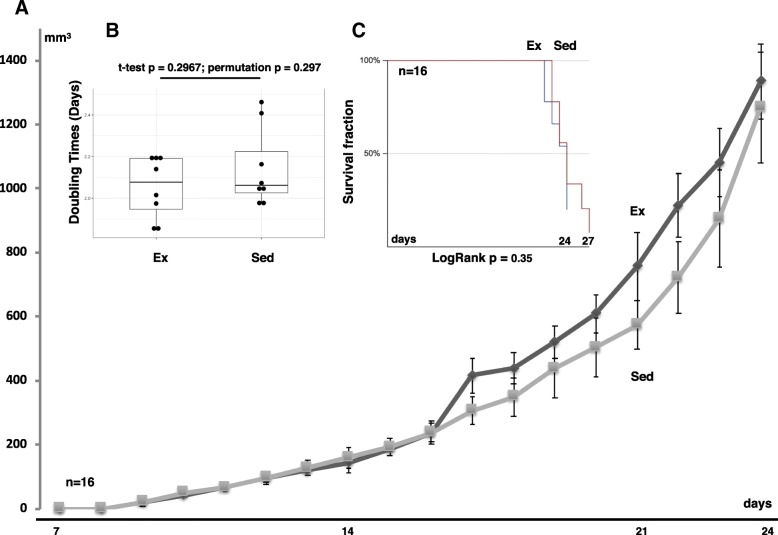
Effects of training on tumor growth and survival times in athymic mice **a** Tumor growth curves for exercise (Ex, *n* = 8) and sedentary (Sed, *n* = 8). Data presented as mean ± SE with 95% confidence intervals. **b** Mean tumor growth rates (doubling times in days). Data presented as mean ± SE with 95% confidence intervals. **c** Kaplan-Meier curve showing no significant difference in survival rates

These results suggest that antitumor immunity, and specifically T cells, are strongly associated with the exercise-mediated suppression of tumor growth. To directly assess this hypothesis, we performed the same endurance training program and tumor growth study with 4 T1 cells on immunodeficient athymic BALB/c mice that do not have mature T cells (Foxn1−/− nude BALB/c, Charles River Labs). Interestingly, there was no statistically significant difference between the exercised and sedentary animals in tumor doubling time (Fig. 5a). This doubling time was indistinguishable from the wild-type sedentary group. Furthermore, in contrast to the wild-type case mean tumor size in the exercised and sedentary athymic mice was never significantly different (Fig. 5b), and the Kaplan Meier survival curve showed no significant difference (Fig. 5c). Finally, no significant difference was detected in mean survival days after inoculation (23.875 days ± 0.6 vs. 22.875 ± 0.5). Together this data suggests that exercise induced an enhanced antitumor immune response that suppressed tumor growth and ultimately enhanced animal survival.

## Discussion

Physical activity has long been thought to mediate prevention of many chronic diseases. Many studies have indicated an association with exercise, physical activity, and/or fitness with tumor incidence, tumor growth, and cancer patient outcomes [32, 33]. Our model for endurance exercise training of murine animals showed a robust effect on the animals themselves but also a significant suppression of tumor growth, consistent with the findings of the studies mentioned above. Most interestingly, our data indicate that exercise promoted an enhanced antitumor immune response, evidenced by an increased CD8^+^/FoxP3^+^ ratio in tumors. This effect of exercise training on the T cell population within tumors has broad implications on the prevention and therapy of solid tumors and gives further evidence that physically fit patients are more likely to ward off chronic disease better than their sedentary counterparts.

The current study indicates an enhanced antitumor immune response in exercised mice but the molecular mechanism by which aerobic exercise could enhance immune function is less clear. Studies have suggested multiple mechanisms that could account for changes in immune function with exercise, such as increased myokines and cytokines secreted by active muscles [34] or an overexpression of adhesion molecules similar to febrile stress [35]. However, there are also other exercise-induced physiological adaptations, and in particular adaptations to hypoxia, that could possibly play a role here, such as enhanced blood and oxygen delivery to tissues. Enhanced blood delivery may possibly suppress a hypoxic response in tumors and lead to a faster degradation of HIF1 *α*, a transcription factor which has been shown to upregulate molecules that attract FoxP3^+^ Treg cells [36, 37]. Investigation is underway to determine molecular mechanisms driving the exercise-induced suppression of intratumoral FoxP3^+^ Tregs.

The development of our novel murine training method also fits an ideal model for testing and quantifying murine exercise. That aerobic exercise is qualitatively conducive to human health is widely accepted. A quantitative dose–response relationship between aerobic exercise and specific health conditions, while likely to exist, remains elusive. This gap in our understanding is due to unclear adherence, uncontrolled treatment fidelity, and the practical and ethical constraints on evaluating these in the critically ill. In order to probe the potential mechanistic pathways that underlie observed effects of endurance exercise on a range of health conditions, it is imperative to develop better exercise rodent models free from adverse and uncontrollable stress. An ideal model must also allow both the control and the quantification of the endurance exercise “dosage”, so that a therapeutic spectrum of efficacy, a clinically effective dose, and predictors of response can be identified.

In the pilot study presented here we have shown that our new forced wheel running model is suitable to induce CEE in rodents without adverse stimuli. Furthermore, notwithstanding that the mice in our model ran significantly *slower* and *less* distance than mice in studies which were based on the voluntary wheel or the electric shock treadmill [4, 8, 38, 39], the lowest-common-denominator dosage we have achieved in continuous running after 8 weeks of training was sufficient to induce significant systemic changes in immune response before and after insult. In addition**,** the dosage “administered” via our exercise model and the training method described in this study are likely more relevant to human exercise routines than those murine exercise models with higher dosages. While exercise rodent models based on voluntary running (simulating HIIT) or electric shock treadmill (inducing CEE with adverse stimuli) are easy to implement (as they require no intervention from the experimenter), they seem inappropriate for achieving better understanding of the elusive dose-response relationship in exercise oncology. Healthy humans, let alone the critically ill, do not run intervals like mice on a voluntary wheel and are not forced to exercise after the point of exhaustion like mice on an electric shock treadmill. Specifically, non-athletes usually can perform HIIT only twice a week, and can do so (with appropriate careful warm-up and cool down interventions) only for 20–30 min, while athletes may increase dosage to 3 times a week [40]. Thus, to truly simulate human-relevant dosage of HIIT, experimenters should block the voluntary wheel after 30 min and allow mice to run only 3 times a week. No such studies have been reported yet, but it is likely that in such conditions the total distance the mice ran would have been shorter and the effects observed would have been dampened. Finally, in contrast to the electric shock treadmill, our long and gentle training protocol got mice to run continuously and perform CEE without incurring stress. The point, however, is that not only such gentle protocol resulted in a more human-relevant dosage, but it was also sufficient to induce the changes we observed.

Finally, the problem of translating murine exercise to human exercise is pertinent to all exercise oncology studies, and we haven’t solved it here. We do believe, however, that our controllable and quantifiable exercise model has a better chance of making progress towards the desired solution. Work is underway to achieve this goal.

## Conclusion

In the present study we have established a controllable and quantifiable rodent model for chronic endurance exercise, developed a low stress, conservative – yet efficacious – training protocol for it, and identified a potential cellular mechanism behind its effect on solid tumor progression. This cellular effect, the suppression of FoxP3^+^ Treg cells recruitment into the tumor, is a novel finding and strongly implicates enhanced antitumor immunity as a means by which aerobic exercise can suppress tumor growth. Furthermore, this result indicates being endurance-trained (i.e. aerobically fit) could increase the likelihood for better patient outcomes and adds to the ever-growing list of reasons for engaging in regular aerobic activity.

## Additional file

Additional file 1: **Figure S1.** Chronic endurance exercise model. **Figure S2.** Running protocol. **Figure S3.** Effect of endurance training on mtDNA copy number in muscle and liver. **Figure S4.** Wild-type animal weight and food intake. **Figure S5.** Effect of endurance training on circulating leukocytes. **Figure S6.** Antitumor immune response in exercised vs. sedentary wild type mice. (PDF 20084 kb)

## Data Availability

The datasets used and/or analyzed during the current study are available from the corresponding author on reasonable request.

## Abbreviations

CBC: Cell Blood Count
CEE: Chronic Endurance Exercise
Ex: Exercised
HIIT: High Intensity Interval Training
IHC: Immunohistochemistry
SE: Standard Error
Sed: Sedentary

## References

[1] Hawley JA, Hargreaves M, Joyner MJ, Zierath JR (2014). Integrative biology of exercise. Cell Metab.

[2] Warburton DE, Nicol CW, Bredin SS (2006). Health benefits of physical activity: the evidence. CMAJ.

[3] Goh J, Ladiges W (2015). Voluntary wheel running in mice. Current protocols in mouse biology.

[4] Marcaletti S, Thomas C, Feige JN (2011). Exercise performance tests in mice. Current protocols in mouse biology.

[5] Legerlotz K, Elliott B, Guillemin B, Smith HK (2008). Voluntary resistance running wheel activity pattern and skeletal muscle growth in rats. Exp Physiol.

[6] Milanovic Z, Sporis G, Weston M (2015). Effectiveness of high-intensity interval training (HIT) and continuous endurance training for VO_2Max_ improvements: a systematic review and meta-analysis of controlled trials. Sports Med.

[7] Foster C, Farland CV, Guidotti F, Harbin M, Roberts B, Schuette J (2015). The effects of high intensity interval training vs. steady state training on aerobic and anaerobic capacity. J Sports Sci Med.

[8] Chen CC, Chang MW, Chang CP, Chan SC, Chang WY, Yang CL (2014). A forced running wheel system with a microcontroller that provides high-intensity exercise training in an animal ischemic stroke model. Braz J Med Biol Res.

[9] Friedenreich CM (2016). Physical activity and cancer outcomes: a precision medicine approach. Clinical Cancer Approach.

[10] Monninkhof EM, Elias SG, Vlems FA (2007). Physical activity and breast cancer: a systematic review. Epidemiol.

[11] Hagar A, Flynn S, Patterson K, Haddad F (2018). Muscular endurance and progression rates of early stage invasive ductal carcinoma – a pilot study. Breast J.

[12] Pedersen BK, Hoffman Goetz L (2000). Exercise and the immune system: regulation, integration, and adaptation. Physiol Rev.

[13] Decker WK, da Silva RF, Sanabria MH, Angelo LS, Guimarães F, Burt BM (2017). Cancer immunotherapy: historical perspective of a clinical revolution and emerging preclinical animal models. Front Immunol.

[14] Tang J, Shalabi A, Hubbard-Lucey VM (2018). Comprehensive analysis of the clinical immuno-oncology landscape. Ann Oncol.

[15] Berendt MJ, North RJ (1980). T-cell-mediated suppression of anti-tumor immunity. JEM..

[16] Shimizu J, Yamazaki S, Sakaguchi S (1999). Induction of tumor immunity by removing CD25+CD4+ T cells: a common basis between tumor immunity and autoimmunity. J Immunol.

[17] Chaudary B, Elkord E (2016). Regulatory T cells in the tumor microenvironment and cancer progression: role and therapeutic targeting. Vaccines..

[18] Facciabene A, Motz GT, Coukos G (2012). T regulatory cells: key players in tumor immune escape and angiogenesis. Cancer Res.

[19] Yan M, Jene N, Byrne D, Millar EK, O’Toole SA, McNeil CM (2011). Recruitment of regulatory T cells is correlated with hypoxia-induced CXCR4 expression and is associated with poor prognosis in basal-like breast cancers. Breast Cancer Res.

[20] Shang B, Liu Y, Jiang S, Liu Y (2015). Prognostic value of tumor-infiltrating FoxP3^+^regulatory T cells in cancers: a systematic review and meta-analysis. Sci Rep.

[21] Carpenter RL, Paw I, Dewhirst MW, Lo HW (2015). Akt phosphorylates and activates HSF-1 independent of heat shock, leading to slug overexpression and epithelial-mesenchymal transition (EMT) of HER2-overexpressing breast cancer cells. Oncogene.

[22] Carpenter RL, Sirkisoon S, Zhu D, Rimkus T, Harrison A, Anderson A (2017). Combined inhibition of AKT and HSF1 suppresses breast cancer stem cells and tumor growth. Oncotarget.

[23] Fitzsimons DP, Diffee GM, Herrick RE, Baldwin KM (1990). Effects of endurance exercise on isomyosin patterns in fast- and slow-twitch skeletal muscles. J Appl Physiol.

[24] Sheng JJ, Jin JP (2015). TNNI1 TNNI2 and TNNI3: evolution, regulation, and protein structure-function relationships. Gene.

[25] Cao X, Zhao ZW, Zhou HY, Chen GQ, Yang HJ (2012). Effects of exercise intensity on copy number and mutations of mitochondrial DNA in gastrocnemus muscles in mice. Mol Med Rep.

[26] Lezi E, Lu J, Burns JM, Swerdlow RH (2013). Effect of exercise on mouse liver and brain bioenergetic infrastructures. Exp Physiol.

[27] Lee HC, Wei YH (2005). Mitochondrial biogenesis and mitochondrial DNA maintenance of mammalian cells under oxidative stress. Int J Biochem Cell Biol.

[28] Goodwin ML, Harris JE, Hernandez A, Gladden B (2007). Blood lactate measurements and analysis during exercise, a guide to clinicians. J Diab Sci & Tech.

[29] Dugger KJ, Chrisman T, Sayner SL, Chastain P, Watson K, Estes R (2018). Beta-2 adrenergic receptors increase TREG cell suppression in an OVA-induced allergic asthma mouse model when mice are moderate aerobically exercised. BMC Immunol.

[30] Horn PL, Pyne DB, Hopkins WG, Barnes CJ (2010). Lower white blood cell counts in elite athletes training for highly aerobic sports. Eur J Appl Physiol.

[31] Thomas RJ, Kenfield SA, Jimenez A (2017). Exercise-induced biochemical changes and their potential influence on cancer: a scientific review. Br J Sports Med.

[32] Moore SC, Lee IM, Weiderpass E, Campbell PT, Sampson JN, Kitahara CM (2016). Association of leisure-time physical activity with risk of 26 types of cancer in 1.44 million adults. JAMA Intern Med.

[33] Friedenreich CM (2016). The role of physical activity in breast Cancer etiology. Semin Oncol.

[34] Pedersen L, Idorn M, Olofsson GH, Lauenborg B, Nookaew I, Hansen RH (2016). Voluntary running suppresses tumor growth through epinephrine- and IL-6-dependent NK cell mobilization and redistribution. Cell Metab.

[35] Evans SS, Repasky EA, Fisher DT (2015). Fever and the thermal regulation of immunity: the immune system feels the heat. Nat Rev Immunol.

[36] Ren L, Yu Y, Wang L, Zhu Z, Lu R, Yao Z (2016). Hypoxia-induced CCL28 promotes recruitment of regulatory T cells and tumor growth in liver cancer. Oncotarget..

[37] Facciabene A, Peng X, Hagemann IS, Balint K, Barchetti A, Wang LP (2011). Tumour hypoxia promotes tolerance and angiogenesis via CCL28 and Treg cells. Nature..

[38] Svensson M, Rosvall P, Boza-Serrano A, Andersson E, Lexell J, Deierborg T (2016). Forced treadmill exercise can induce stress and increase neuronal damage in a mouse model of global cerebral ischemia. Neurobiology of Stress.

[39] Meijer JH, Robbers Y (2014). Wheel running in the wild. Proc R Soc B.

[40] Karlsen T, Aamot I, Haykowsky M, Rognmo O (2017). High intensity interval training for maximizing health outcomes. Prog Cardiovasc Dis.

