# Supplementary material for: Endurance training slows breast tumor growth in mice by suppressing Treg cells recruitment to tumors
Source: BMC Cancer. 2019 Jun 4;19:536. doi: 10.1186/s12885-019-5745-7 (PMC6549262; doi:10.1186/s12885-019-5745-7)
Supplement: Supplementary file 1 — Figure S1. Chronic endurance exercise model. Figure S2. Running protocol. Figure S3. Effect of endurance training on mtDNA copy number in muscle and liver. Figure S4. Wild-type animal weight and food intake. Figure S5. Effect of endurance training on circulating leukocytes. Figure S6. Antitumor immune response in exercised vs. sedentary wild type mice. (PDF 20084 kb) [file 12885_2019_5745_MOESM1_ESM.pdf]

A

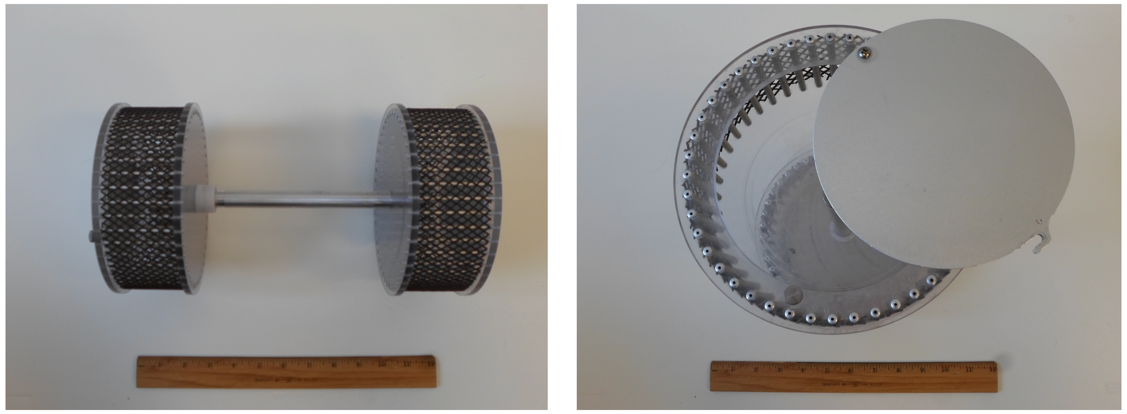

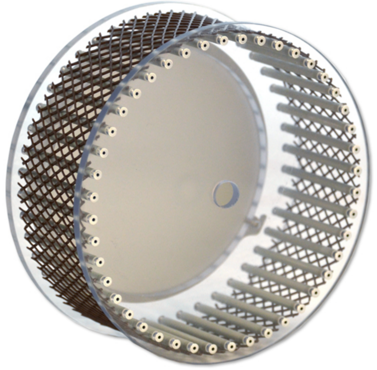

Weight: 360gr (0.8lb)
Wheel Diameter: 15.1cm (5.94”) ID
Wheel Diameter: 17.1cm (6.75”) OD
Wheel Width: 5.7cm (2.25”) (int.)
Wheel Width: 7cm (2.75”) (ext.)

B

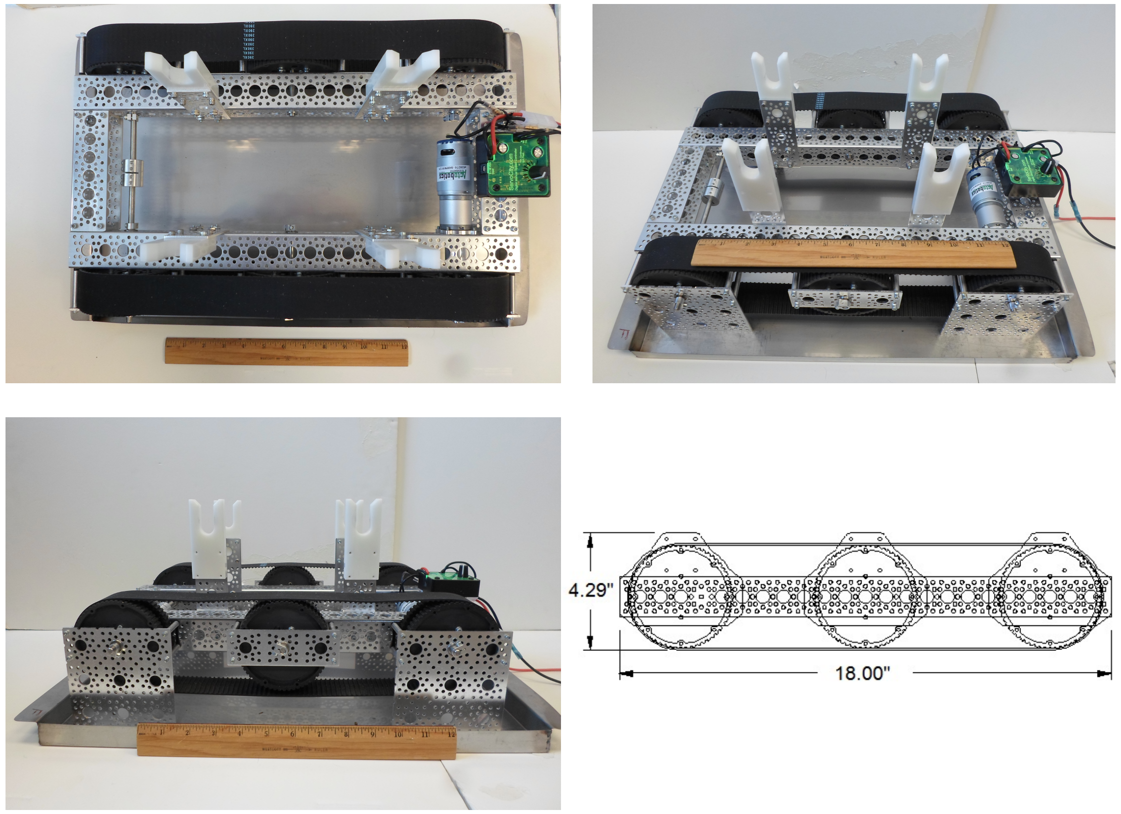

C

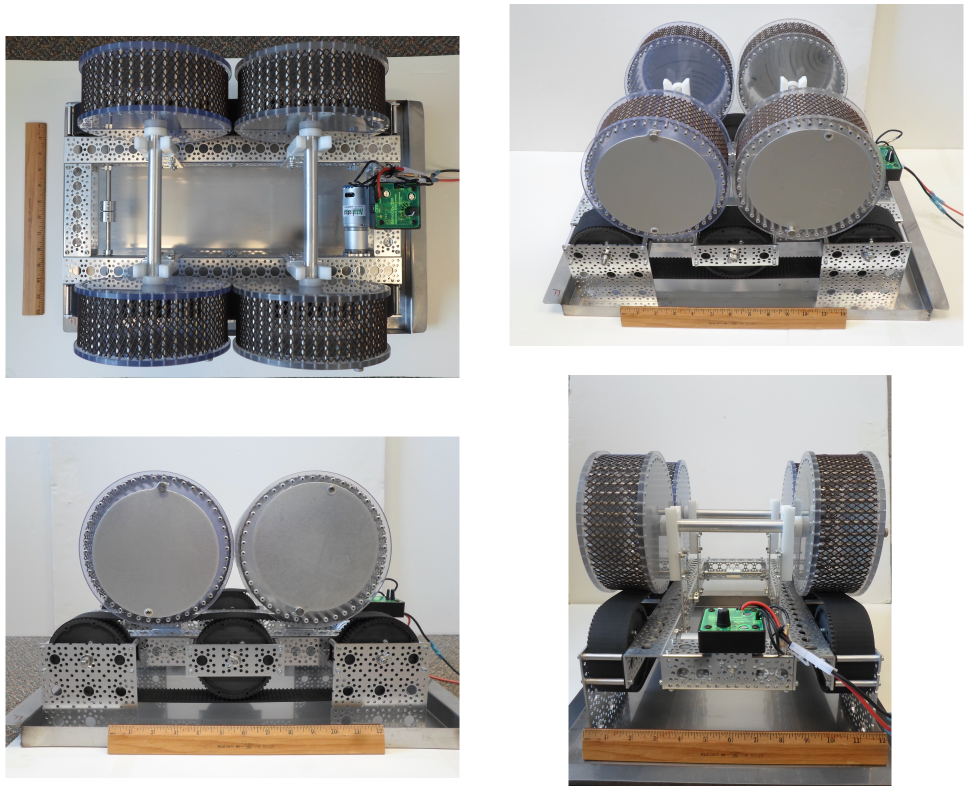

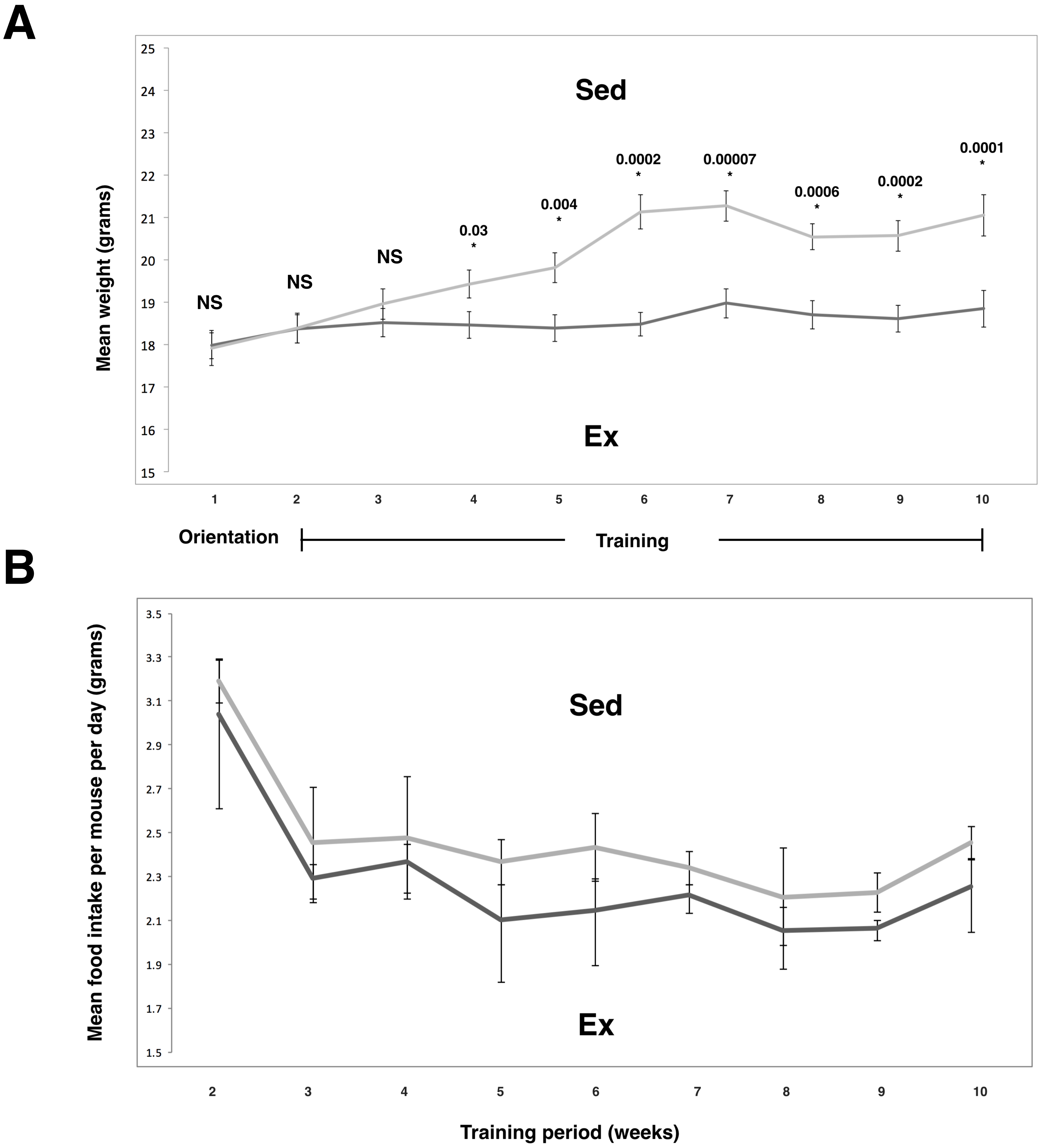
A
Sed
Ex
Mean weight (grams)
25
24
23
22
21
20
19
18
17
16
15
NS
NS
NS
0.03
*
0.004
*
0.0002
*
0.00007
*
0.0006
*
0.0002
*
0.0001
*
1
2
3
4
5
6
7
8
9
10
Orientation
Training
B
Sed
Ex
Mean food intake per mouse per day (grams)
3.5
3.3
3.1
2.9
2.7
2.5
2.3
2.1
1.9
1.7
1.5
2
3
4
5
6
7
8
9
10
Training period (weeks)

Week	Weekday	min		m/min	m
1	mon	10	10	4	40
	tue	10	10	4	40
	wed	10	10	4	40
	thu	10	10	4	40
	fri	10	10	4	40
2	mon	14	7<br>7	6<br>8	98
	tue	14	7<br>7	6<br>8	98
	wed	14	5<br>9	6<br>8	102
	thu	14	5<br>9	6<br>8	92
	fri	14	7<br>4<br>3	6<br>6<br>8	92
3	mon	16	8<br>5<br>3	6<br>8<br>10	118
	tue	16	8<br>5<br>3	6<br>8<br>10	118
	wed	16	8<br>5<br>3	6<br>8<br>10	118
	thu	16	8<br>5<br>3	6<br>8<br>10	118
	fri	16	8<br>5<br>3	6<br>8<br>10	118
4	mon	18	10<br>6<br>2	6<br>8<br>10	128
	tue	18	5<br>10<br>3	6<br>8<br>10	128
	wed	18	6<br>9<br>3	6<br>8<br>10	140
	thu	18	5<br>10<br>3	6<br>8<br>10	138
	fri	18	3<br>11<br>4	6<br>8<br>10	140
5	mon	20	4<br>6<br>8<br>2	6<br>8<br>10<br>12	172
	tue	20	4<br>5<br>9<br>2	6<br>8<br>10<br>12	156
	wed	20	4<br>8<br>7<br>1	6<br>8<br>10<br>12	161
	thu	20	4<br>6<br>8<br>2	6<br>8<br>10<br>12	164
	fri	20	4<br>5<br>9<br>2	6<br>8<br>10<br>12	164
6	mon	22	4<br>6<br>10<br>2	6<br>8<br>10<br>12	196
	tue	22	4<br>4<br>12<br>2	6<br>8<br>10<br>12	200
	wed	22	4<br>4<br>12<br>2	6<br>8<br>10<br>12	196
	thu	22	3<br>4<br>13<br>2	6<br>8<br>10<br>12	204
	fri	22	2<br>4<br>14<br>2	6<br>8<br>10<br>12	208
7	mon	24	2<br>4<br>16<br>2	6<br>8<br>10<br>12	228
	tue	24	2<br>4<br>16<br>2	6<br>8<br>10<br>12	228
	wed	24	2<br>3<br>17<br>2	6<br>8<br>10<br>12	230
	thu	24	1<br>3<br>18<br>2	6<br>8<br>10<br>12	234
	fri	24	1<br>3<br>18<br>2	6<br>8<br>10<br>12	234
8	mon	26	2<br>4<br>18<br>2	6<br>8<br>10<br>12	248
	tue	26	1<br>4<br>19<br>2	6<br>8<br>10<br>12	252
	wed	26	1<br>3<br>20<br>2	6<br>8<br>10<br>12	254
	thu	26	1<br>2<br>21<br>2	6<br>8<br>10<br>12	256
	fri	26	1<br>1<br>22<br>2	6<br>8<br>10<br>12	258
					6189

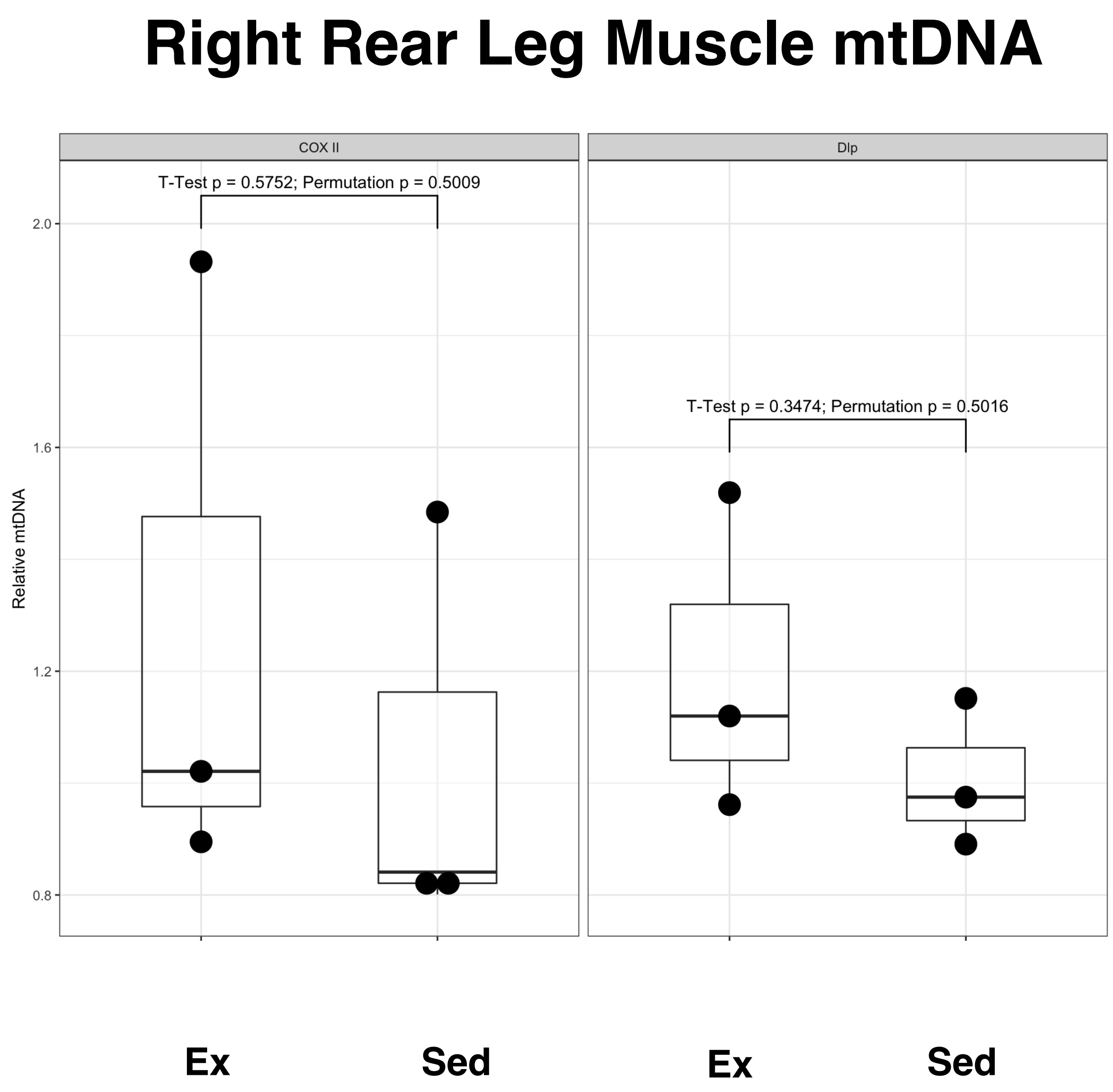
Right Rear Leg Muscle mtDNA
COX II
Dlp
T-Test p = 0.5752; Permutation p = 0.5009
T-Test p = 0.3474; Permutation p = 0.5016
Relative mtDNA
2.0
1.6
1.2
0.8
Ex
Sed
Ex
Sed

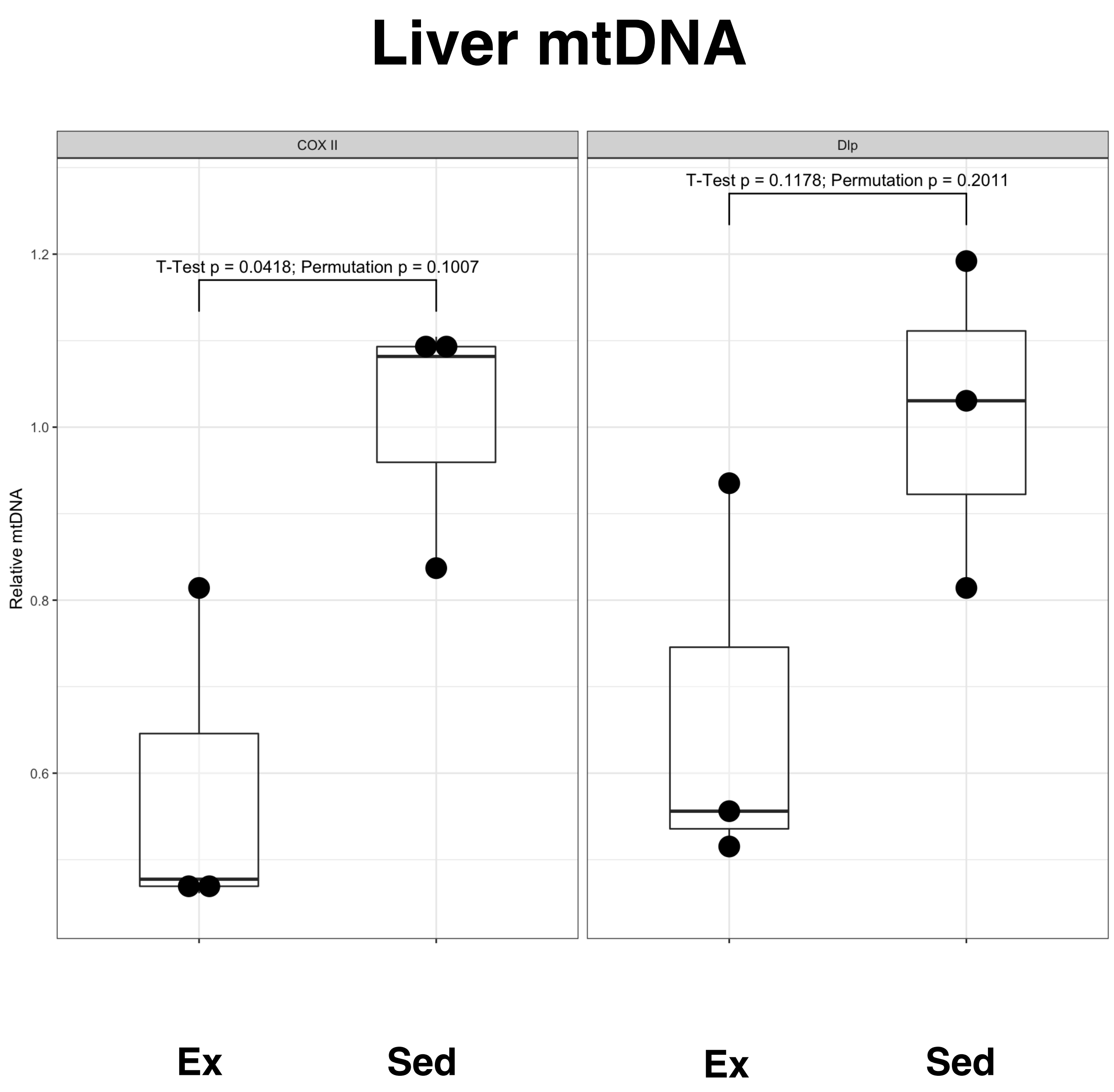
Liver mtDNA
COX II
Dlp
T-Test p = 0.0418; Permutation p = 0.1007
T-Test p = 0.1178; Permutation p = 0.2011
Relative mtDNA
1.2
1.0
0.8
0.6
Ex
Sed
Ex
Sed

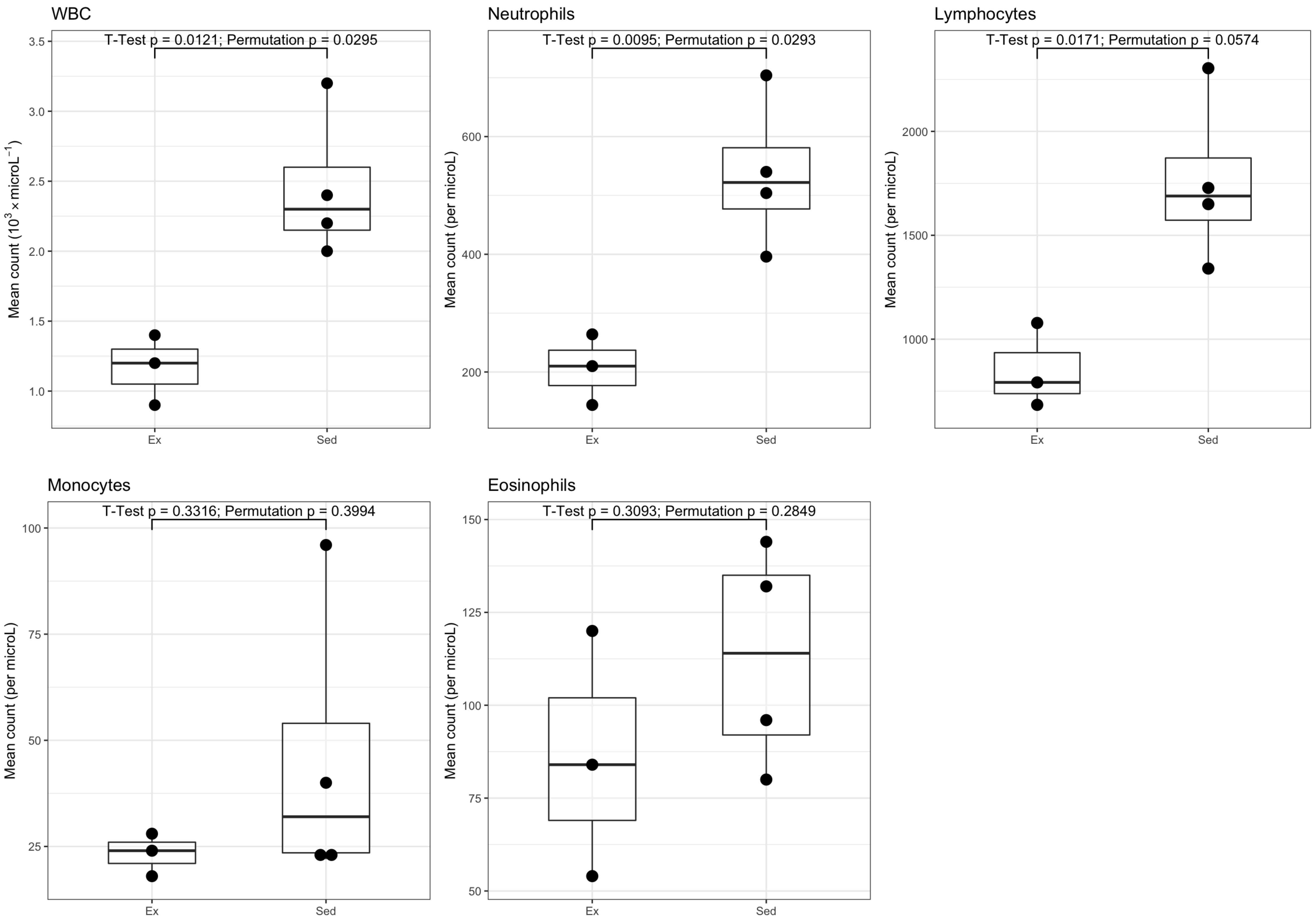
WBC
T-Test p = 0.0121; Permutation p = 0.0295
Mean count ($10^3 \times$ microL^{-1})
Neutrophils
T-Test p = 0.0095; Permutation p = 0.0293
Mean count (per microL)
Lymphocytes
T-Test p = 0.0171; Permutation p = 0.0574
Mean count (per microL)
Monocytes
T-Test p = 0.3316; Permutation p = 0.3994
Mean count (per microL)
Eosinophils
T-Test p = 0.3093; Permutation p = 0.2849
Mean count (per microL)
Ex
Sed

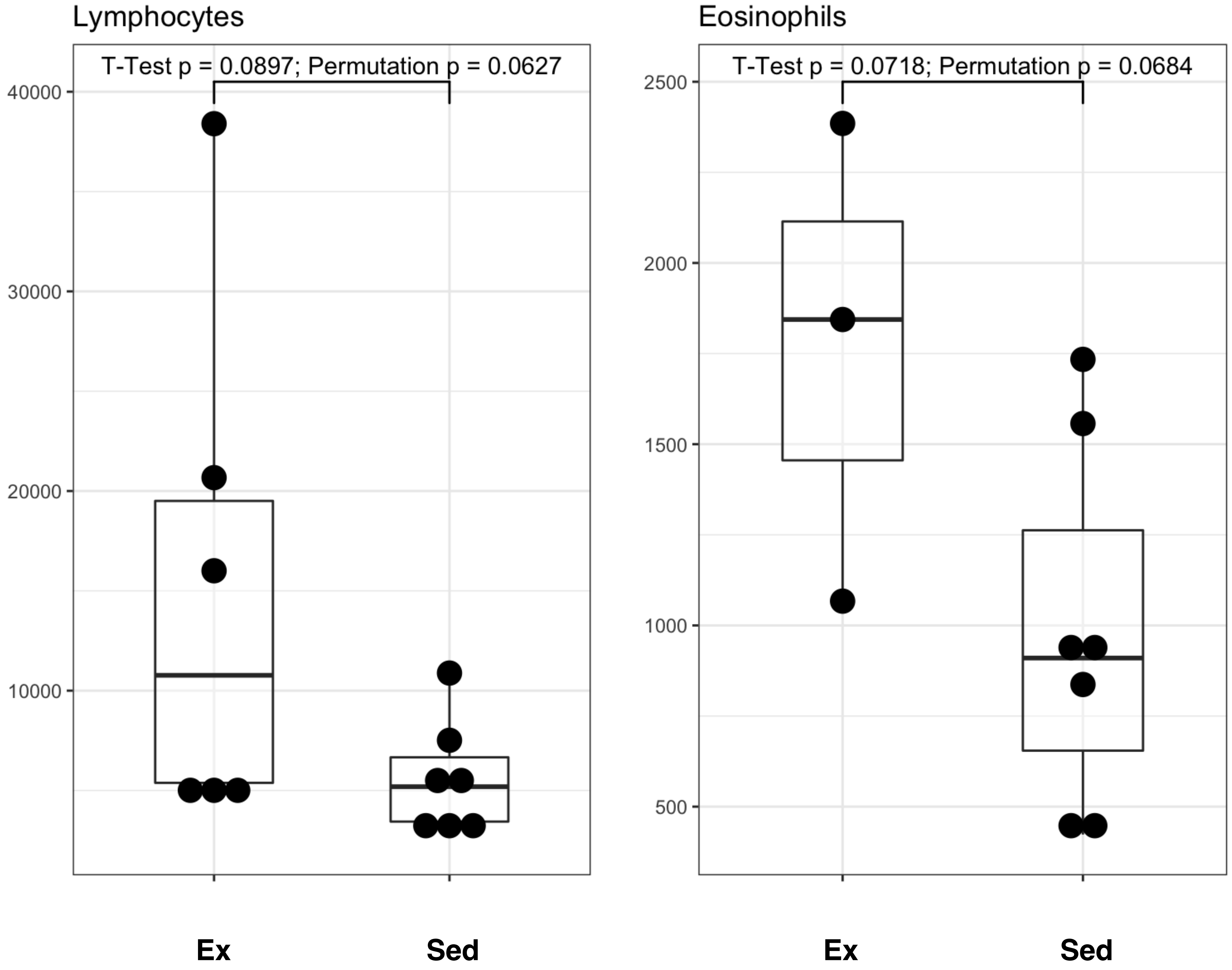
Lymphocytes
T-Test p = 0.0897; Permutation p = 0.0627
40000
30000
20000
10000
Ex
Sed
Eosinophils
T-Test p = 0.0718; Permutation p = 0.0684
2500
2000
1500
1000
500
Ex
Sed
